# Using neighborhood gray tone difference matrix texture features on dual time point PET/CT images to differentiate malignant from benign FDG-avid solitary pulmonary nodules

**DOI:** 10.1186/s40644-019-0243-3

**Published:** 2019-08-16

**Authors:** Song Chen, Stephanie Harmon, Timothy Perk, Xuena Li, Meijie Chen, Yaming Li, Robert Jeraj

**Affiliations:** 1 Department of Nuclear Medicine, The First Hospital of China Medical University, No.155 North Nanjing Street, Heping District, Shenyang City, Liaoning Province 110001 People’s Republic of China; 2 Department of Medical Physics, School of Medicine and Public Health, University of Wisconsin-Madison, Madison, USA

**Keywords:** Positron emission tomography computed tomography, Fluorodeoxyglucose F18, Solitary pulmonary nodules, Lung neoplasms

## Abstract

**Objective:**

Lung cancer usually presents as a solitary pulmonary nodule (SPN) on diagnostic imaging during the early stages of the disease. Since the early diagnosis of lung cancer is very important for treatment, the accurate diagnosis of SPNs has much importance. The aim of this study was to evaluate the discriminant power of dual time point imaging (DTPI) PET/CT in the differentiation of malignant and benign FDG-avid solitary pulmonary nodules by using neighborhood gray-tone difference matrix (NGTDM) texture features.

**Methods:**

Retrospective analysis was carried out on 116 patients with SPNs (35 benign and 81 malignant) who had DTPI ^18^F-FDG PET/CT between January 2005 and May 2015. Both PET and CT images were acquired at 1 h and 3 h after injection. The SUV_max_ and NGTDM texture features (coarseness, contrast, and busyness) of each nodule were calculated on dual time point images. Patients were randomly divided into training and validation datasets. Receiver operating characteristic (ROC) curve analysis was performed on all texture features in the training dataset to calculate the optimal threshold for differentiating malignant SPNs from benign SPNs. For all the lesions in the testing dataset, two visual interpretation scores were determined by two nuclear medicine physicians based on the PET/CT images with and without reference to the texture features.

**Results:**

In the training dataset, the AUCs of delayed busyness, delayed coarseness, early busyness, and early SUV_max_ were 0.87, 0.85, 0.75 and 0.75, respectively. In the validation dataset, the AUCs of visual interpretations with and without texture features were 0.89 and 0.80, respectively.

**Conclusion:**

Compared to SUV_max_ or visual interpretation, NGTDM texture features derived from DTPI PET/CT images can be used as good predictors of SPN malignancy. Improvement in discriminating benign from malignant nodules using SUVmax and visual interpretation can be achieved by adding busyness extracted from delayed PET/CT images.

**Electronic supplementary material:**

The online version of this article (10.1186/s40644-019-0243-3) contains supplementary material, which is available to authorized users.

## Introduction

A solitary pulmonary nodule (SPN) is defined radiologically as an intraparenchymal lung lesion of less than 3 cm in diameter, with no associated atelectasis or adenopathy [1]. Since SPNs may indicate malignant disease, the management of SPNs is clinically controversial and mainly dependent on the perceived probability of malignancy. The causes of SPNs range from malignancy, such as primary lung cancer or metastatic cancer sites, to inflammation and other benign diseases. Previous studies have shown that SPNs are detected in almost 70% of subjects receiving low-dose CT-based lung cancer screenings [2], whereas another study found that 53% of detected SPNs were characterized as malignant nodules [3]. Lung cancer usually presents as an SPN on diagnostic imaging during early stages of the disease [1]. Since the early diagnosis of lung cancer is very important for treatment, as it would allow surgical resection to increase survival rates, the accurate diagnosis of SPNs has even more importance.

^18^F-FDG PET/CT imaging has greatly contributed to the differentiation of benign and malignant SPNs. In general, a standardized uptake value (SUV) greater than 2.5 g/ml is suggestive of malignancy [4]. However, many reports have reported false positive results in infectious lung diseases such as granuloma, tuberculosis, or pneumonitis. These infectious diseases also exhibit high uptake of FDG, especially tuberculosis [4]. This causes the specificity of PET/CT in granuloma-endemic regions to be much lower than that of nonendemic regions [5, 6]. To enhance the diagnostic accuracy of FDG PET/CT, some researchers suggest using dual time point imaging (DTPI) PET/CT [7]. Previous studies using an SUV threshold of 10% of the retention index have shown that the retention index increased between the early and delayed scans and improved the accuracy of FDG-PET [8, 9]. In China, which has the second largest incidence of tuberculosis, delayed time point imaging is commonly used by nuclear medicine physicians to differentiate malignant from benign solitary pulmonary nodules. However, after years of clinical validation, physicians found that the retention index of DTPI had no additional value in differentiating between malignant and benign lung nodules, especially in FDG-avid lesions [4, 10]. This suggests that simple uptake metrics, such as maximum uptake (SUV_max_) and retention index, may not be sufficient for differentiating malignant from benign SPNs.

Our previous study demonstrated that benign and malignant SPNs can be differentiated by using machine learning models trained by including a large number of texture features from PET/CT images [11]. The results of that study suggested that texture features derived from a neighborhood gray tone difference matrix (NGTDM) might be useful for discriminating malignant SPNs from benign SPNs.

In this paper, we used texture features derived from a NGTDM to classify malignant nodules in a patient from granuloma-endemic regions. Based on the definition of those NGTDM textural features, the value of those texture features reflected the intensity differences between a voxel and its neighboring voxels [12]. We hypothesized that NGTDM texture features extracted from DTPI PET/CT images might be good predictors for malignant solitary pulmonary nodules.

## Materials and methods

### Subjects

Between January 2005 and May 2015, 177 subjects with an identified SPN on ^18^F-FDG DTPI PET/CT imaging at a single center were retrospectively reviewed. The diagnosis of malignant lesions was confirmed by pathology results. The diagnosis of benign lesions was confirmed by pathology review or follow-up imaging after at least 12 months. In accordance with work carried out by Orlhac et al. showing that PET texture features were not reliable in small lesions [13], we excluded lesions with a metabolic volume smaller than 64 voxels and lesions with SUV_max_ smaller than 2.5 from this study, which left 116 subjects for analysis. Of these patients, 81 were male and 35 were female, and the mean age was 60.20 ± 11.23 years. Eighty-one lesions were malignant nodules, and 35 lesions were benign. The final diagnosis and subtypes of the nodules are summarized in Table 1.

**Table 1:** SPNs: final diagnosis and subtypes

Type	Diagnosis	Number of cases
Benign		35
	Active tuberculosis	9
	Granuloma	8
	Inflammatory pseudotumor	2
	Non-specific inflammation	1
	Parasite	1
	Reduced nodules	9
	Stable nodules	5
Malignant		81
	Adenocarcinoma	45
	Large Cell Carcinoma	2
	Mucoepidermoid carcinoma	2
	NSCLC	9
	Sarcomatoid carcinoma	1
	SCLC	11
	Squamous cell carcinoma	10
	Thymic carcinoma	1

An eleven-fold cross-validation was used to divide the study cohort into training data and testing data. By using 11-fold cross-validation, the original cohort was randomly divided into 11 similar sized subgroups. Of the 11 subgroups, a single subgroup was retained as the testing data for visual interpretations, and the remaining 10 subgroups were used as training data to calculate the threshold for each texture feature to diagnose malignant nodules. The cross-validation process was then repeated 11 times, with each of the 11 subgroups used exactly once as the testing data. The 11 results from each subgroup were then averaged to produce the final results.

### PET/CT imaging

The patients fasted at least 4~6 h before ^18^F-FDG injection. The blood glucose level was checked immediately before injection. All scans were obtained on a GE Discovery LS 4 PET/CT scanner. Early and delayed PET/CT images were acquired at approximately 60 min and 180 min after injection of 5.55 MBq/kg ^18^F-FDG. Both early and delayed PET imaging was acquired for 3 min per bed position in 2D mode. Neither motion correction nor breath gating were performed. PET images were reconstructed, using ordered-subsets expectation maximization with 2 iterations, 28 subsets and an 8-mm Gaussian filter, into a 128 × 128 matrix with 4.25 mm/slice. CT scanning was performed under the following parameters: 120 kV; auto exposure; 512 × 512 matrix; and free breathing. The PET/CT scanner was calibrated, and daily QCs were performed.

### Texture feature extraction

All nodules were segmented using 3D-slicer (ver. 4.4.0) [14] without knowledge of the clinical data by the consensus of two experienced nuclear medicine physicians. Three neighborhood gray-tone difference matrix-based texture features (coarseness, contrast, and busyness) were extracted. To calculate the texture features, a binning process with 128 bin size was used, then a 5 × 5 × 5 voxel sub volume was extracted around each voxel in the lesion, and the features were computed on each directional plane (axial, sagittal, and coronal) and then averaged over the three planes to obtain the feature value for that voxel. The texture feature values for each lesion were calculated as the average feature values of all the voxels within the lesion [11, 15, 16].

For all the lesions in the training dataset, receiver operating characteristics (ROC) analysis was performed on each texture feature to differentiate malignant from benign nodules. Values plotted nearest the upper left corner of the ROC plot were considered to be the optimal operating point for that feature.

### Image interpretation

All nodules were evaluated visually without knowledge of the clinical data by two experienced nuclear medicine physicians. Visual interpretations were performed on the integration of CT characteristics (attenuation, shape, margin, and size), PET characteristics (uptake degree, distribution of uptake, spatial volume effect and SUV_max_) and the changes in uptake on the delayed PET images [17]. A 5-point scale interpretation score was made for each lesion based on the likelihood of the lesion being benign or malignant (1, definitely benign; 2, likely benign; 3, equivocal; 4, likely malignant; 5, definitely malignant). If the interpretation scores were discordant between two readers, they met to form a consensus.

After visual interpretations, the physicians gave another 5-point scale interpretation score for each nodule. At this time, the physicians knew the value of each texture feature, the discriminant power of each texture feature and the threshold value for each texture feature that we calculated in the training dataset. Based on the integration of image characteristics and the value of each texture feature, the physicians gave another 5-point scale interpretation score for each nodule.

### Statistics

Receiver operating characteristics (ROC) analysis was performed on each index (texture features, SUV_max_, visual interpretation scores). The discriminant power of each index was evaluated using the areas under the ROC curves (AUC). Values plotted nearest the upper left corner of the ROC plot were considered to be the optimal threshold. The diagnostic accuracy, sensitivity, and specificity were calculated using the optimal threshold. The Pearson correlations test was performed to evaluate the correlation relationship between the visual interpretation scores and each index. The Wilcoxon rank sum test was applied to evaluate significant differences in those features for malignant and benign lesions.

All statistical analyses were performed using SPSS 17.0 software or MATLAB 2013b software. *P* < 0.05 was considered to indicate statistical significance.

## Results

### The distribution of texture features in benign and malignant SPNs

The Wilcoxon rank sum test showed that busyness and coarseness were significantly different between benign and malignant lesions on both early time-point PET images and delayed time-point PET images (Table 2). In addition to the early contrast, all the other texture features were significantly different between benign and malignant lesions. Benign lesions had a higher busyness value and a lower coarseness value in both early time-point PET images and delayed time-point PET images.

**Table 2:** Results of Wilcoxon rank sum test

Features	Malignant lesions^a^	Benign lesions^a^	Z value	*P* value
Early Busyness	(4.28 ± 0.38)*10^− 2^	(4.71 ± 0.57)*10^− 2^	−4.213	*p* < 0.01
Early Coarseness	(8.04 ± 1.52)*10^− 2^	(6.91 ± 1.42)*10^− 2^	−3.55	*p* < 0.01
Early Contrast	(1.07 ± 0.33) *10^3^	(1.07 ± 0.29) *10^3^	−0.28	*P* = 0.78
Delayed Busyness	(4.34 ± 0.40) *10^−2^	(4.89 ± 0.42)*10^− 2^	−6.02	*p* < 0.001
Delayed Coarseness	(8.06 ± 1.29)*10^−2^	(6.50 ± 1.07)*10^−2^	−5.56	*p* < 0.001
Delayed Contrast	(1.09 ± 0.29)*10^3^	(1.22 ± 0.38) *10^3^	−2.03	*p* = 0.042
Early SUV_max_	11.22 ± 6.24	6.94 ± 3.58	−4.11	*p* < 0.01

^a^ Data: Mean ± SD

### Comparison of discriminant power of texture features and SUV_max_

**Fig. 1:**
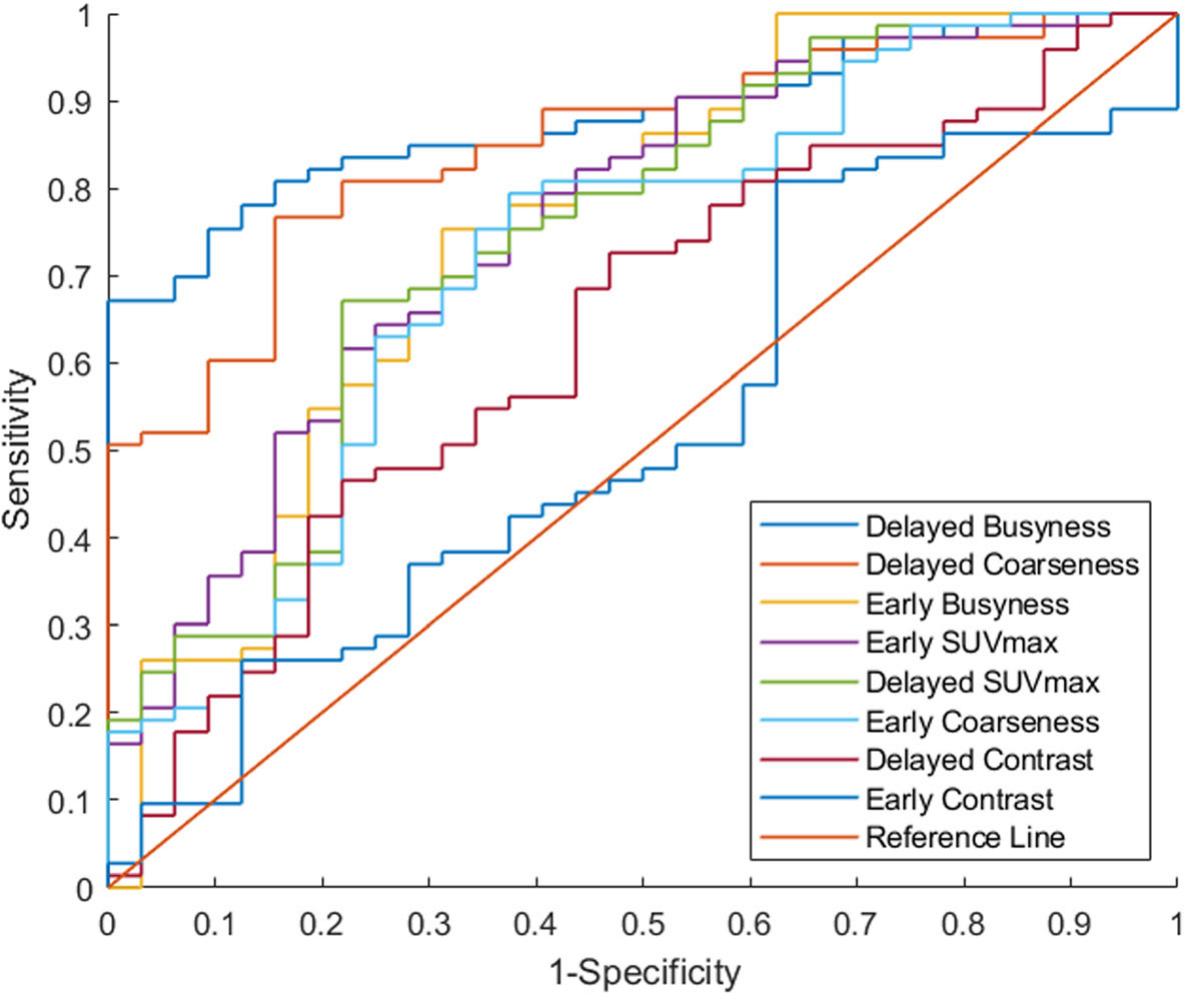
ROC curves of texture features, early SUV_max_ and delayed SUV_max_. AUC showed the ability of texture features, early SUV_max_ and delayed SUV_max_ to distinguish malignant from benign SPNs

The discriminant power of each index can be compared by the AUC of ROC curves (Fig. 1). The AUC of early SUV_max_ and delayed SUV_max_ were 0.75 and 0.74, respectively (Table 3). The delayed busyness and delayed coarseness had greater discriminant power than early SUV_max_ (Table 3). By using the optimal threshold, delayed busyness outperformed other features and achieved the best accuracy, sensitivity, and specificity (Table 3).

**Table 3:** The area under the receiver operating characteristics for each feature

Features	AUC^a^	Threshold	Sensitivity	Specificity	Accuracy
Delayed Busyness	0.87	0.0460	0.81	0.84	0.82
Delayed Coarseness	0.85	0.0726	0.77	0.84	0.79
Early Busyness	0.75	0.0446	0.75	0.68	0.73
Early SUV_max_	0.75	8.43	0.64	0.75	0.68
Delayed SUV_max_	0.74	11.42	0.67	0.78	0.70
Early Coarseness	0.72	0.0696	0.75	0.66	0.72
Delayed Contrast	0.63	1154.91	0.68	0.56	0.65
Early Contrast	0.52	1331.21	0.80	0.38	0.68

^a^ *AUC* The area under the receiver operating characteristicss

### Results of visual interpretation

Two physicians visually interpreted all lesions. Without reference to texture features, among 81 malignant lesions, the physicians correctly diagnosed 73 lesions (90.1%) as definitely or likely malignant lesions and misclassified 4 lesions (5%) as likely benign or definitely benign lesions. Adding texture features as a predictor for malignant lesions, 3 out of 4 equivocal lesions were reclassified as likely malignant lesions. Without reference to texture features, among 35 benign lesions, the physicians correctly classified 12 lesions (34.29%) as definitely or likely benign lesions and 10 lesions (28.57%) as equivocal, and they misclassified 13 lesions (37.14%) as malignant lesions. Adding texture as a reference, 6 out of 8 likely benign lesions were reclassified as definitely benign lesions, 6 out of 10 equivocal lesions were reclassified as likely benign lesions, 3 out of 5 likely malignant lesions were reclassified as equivocal, and 5 out of 8 definitely malignant lesions were reclassified as likely malignant lesions (Table 4).

**Table 4:** The results of visual interpretation with and without texture features

Visual interpretation with texture features		Visual interpretation without texture features				
		Definitely Benign	Likely Benign	Equivocal	Likely Malignant	Definitely Malignant
Benign Nodules	Definitely benign	4	6			
	Likely benign		1	6		
	Equivocal		1	4	3	
	Likely malignant				2	5
	Definitely malignant					3
Malignant Nodules	Definitely benign	1	2			
	Likely benign		1			
	Equivocal			1	1	
	Likely malignant			3	1	10
	Definitely malignant				10	51

The Pearson correlation test (Table 5 and Additional file 1) showed that: except early contrast, texture features were significantly correlated with visual interpretation scores. The delayed texture features had higher correlation coefficients than early texture features.

**Table 5:** The Pearson correlation coefficients of texture features and visual interpretation score

	Early SUVmax	Early Busyness	Early Contrast	Early Coarseness	Delayed SUVmax	Delayed Busyness	Delayed Coarseness	Delayed Contrast
Visual interpretation with texture features	0.381^*^	0.629^*^	0.143	0.520^*^	0.419^*^	0.740^*^	0.681^*^	0.345^*^
Visual interpretation without texture features	0.308^*^	0.575^*^	0.130	0.461^*^	0.353^*^	0.638^*^	0.596^*^	0.325^*^

* Pearson test showed that the *P* value was below 0.01

**Fig. 2:**
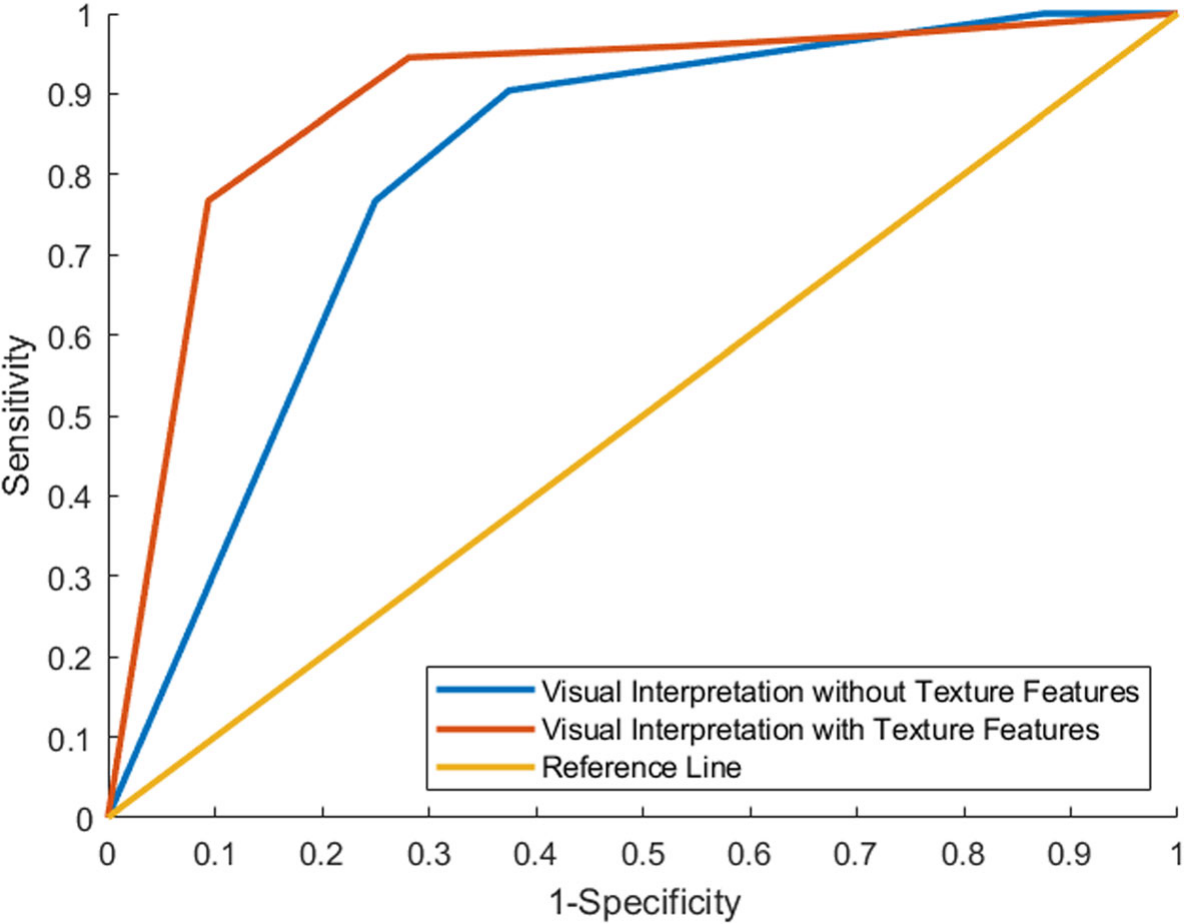
ROC curves of physicians’ visual interpretations with and without texture features. AUC showed that with the help of texture features, physicians performed better in differentiating malignant from benign lesions. By employing the best performance threshold, visual interpretation with reference to the texture features obtained 76.71, 90.63 and 80.91% sensitivity, specificity, and accuracy, respectively. By employing the best performance threshold, visual interpretation without reference to the texture features obtained 76.71, 75.00 and 76.20% sensitivity, specificity, and accuracy, respectively

The AUCs of the visual interpretations with and without texture features were 0.80 and 0.89, respectively (Fig. 2). AUCs showed that with the help of texture features, the physicians performed better in differentiating malignant from benign lesions. By employing the best performance threshold, visual interpretation with reference to the texture features had higher specificity (90.63%) than interpretation without reference to the texture features (75.00%).

## Discussion

This study demonstrated that quantitative NGTDM texture features derived from dual time point PET/CT images were good predictors for diagnosing malignant SPNs in patients from granuloma-endemic regions. In these regions, busyness extracted from delayed PET images offered a greater discriminatory power, marked by higher accuracy, specificity, and sensitivity, than commonly used clinical metrics (early SUV_max_). With the help of NGTDM texture features, the physicians performed better in differentiating malignant SPNs from benign SPNs. To our knowledge, this is the first report to evaluate the performance of delayed NGTDM texture features for the diagnosis of solitary pulmonary nodules. The enhancement in discriminatory performance shown in this study could benefit patients by preventing the high false positive rate of PET/CT for granuloma-endemic regions.

^18^F-FDG PET/CT has been widely used for SPN diagnosis. However, the specificity of FDG PET/CT is lower in granuloma-endemic regions than in nonendemic regions [5, 6]. Some benign lesions, such as tuberculosis and granuloma, also have increased ^18^F-FDG uptake in PET, leading to false-positive results. In this study, 21 of the FDG-avid (SUV_max_ > 2.5) benign lesions were diagnosed with physiology results, and 80.95% (17/21) of them were tuberculosis or granuloma. This suggested that using SUV_max_ as the only index for diagnosis leads to poor differentiation of malignant nodules from tuberculosis and granuloma, which is similar to what previous studies reported [5, 18].

Multiple guidelines for pulmonary cancer or management of pulmonary nodules have suggested a biopsy test for a single solid pulmonary nodule with a diameter larger than 8 mm, especially those nodules with high suspicion of lung cancer in PET/CT images [19–22]. In this study, all the nodules we studied were FDG-avid with SUV_max_ > 2.5 g/ml. According to the Pulmonary Nodules Guidelines for Asia [21], any solid nodule with SUV > 2.5 g/ml was strongly recommended for surgical biopsy, and similar recommendations were found in the NCCN guidelines for NSCLC [22] and lung cancer screening [20]. According to those guidelines, 30% (35/116) of the patients in this study would receive excessive surgical resection for benign nodules. As 25% of those patients were pathologically diagnosed with tuberculosis, this would lead to a higher complication rate for fistulas and infection. By referencing the texture features, the AUC improved from 0.80 to 0.89, and 31.42% (11/35) patients with benign lesions were diagnosed correctly. This would have prevented these patients from receiving excessive surgical resections.

In the training dataset, delayed busyness and delayed coarseness showed much higher AUC (0.87 and 0.85, respectively) than early SUV_max_ (0.75). The diagnostic abilities of delayed busyness and delayed coarseness are better than early SUV_max_, with higher accuracy, sensitivity, and specificity. Therefore, busyness and coarseness extracted from the delayed time point for FDG PET/CT are good predictors of malignant SPNs and might be a semiautomated quantitative tool to supplement other patient information for physicians.

**Fig. 3:**
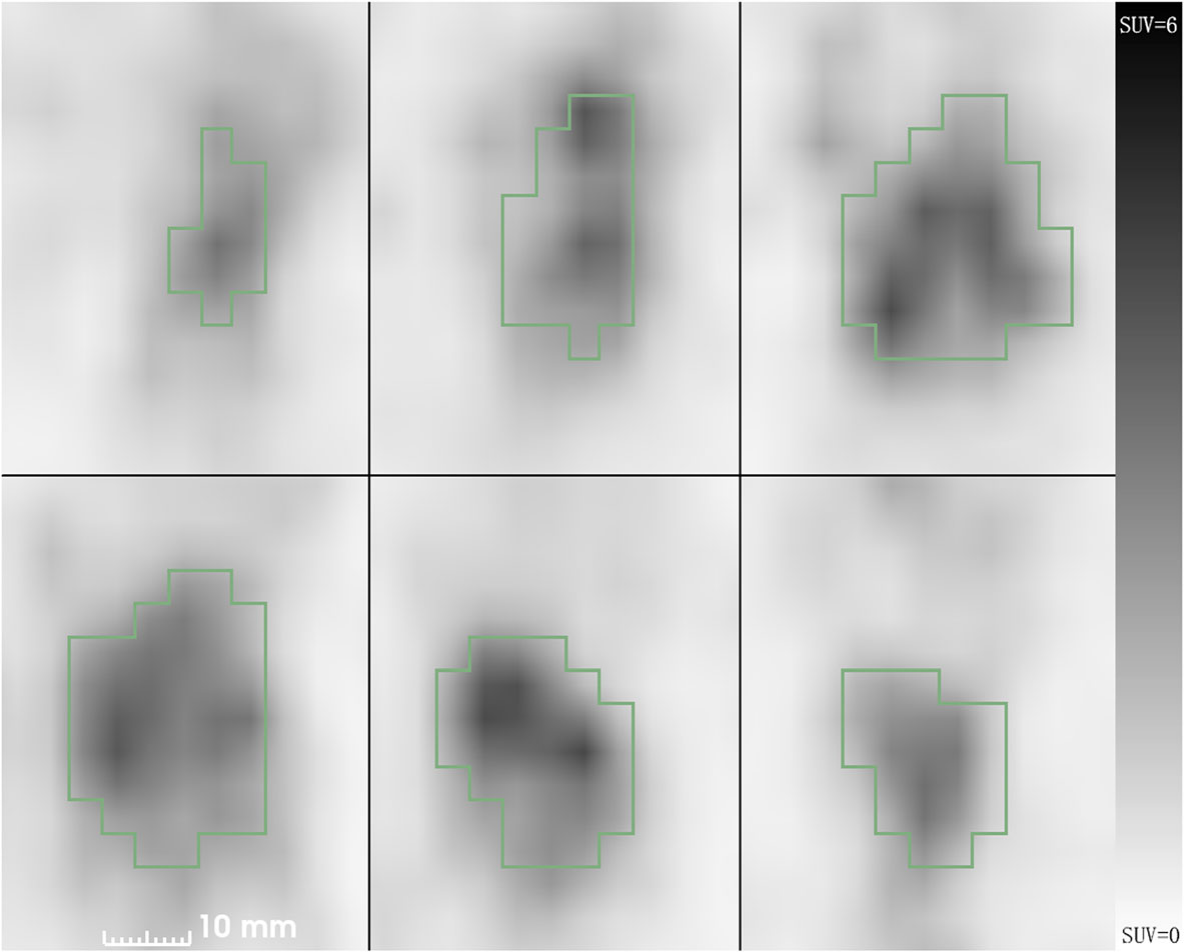
Delayed PET images of a reduced nodule are shown. The high uptake regions of the lesion are separated spatially, which leads to a higher busyness value and a lower coarseness value (delayed SUVmax = 5.80, delayed busyness = 5.98*10^−2^, delayed coarseness = 4.41*10^− 2^, delayed cluster prominence = 2.65*10^8^)

Both busyness and coarseness are calculated from the neighborhood gray-tone difference matrix. Busyness describes the spatial frequency of intensity changes, with lesions exhibiting high busyness indicating high spatial frequency of intensity changes within the lesion [12]. Our hypothesis is that there might be multiple origins of inconsistency in spatial intensity for benign lesions, including bacterium infection, edema cells, presence of bleeding, obstructed bronchioles, and fibrotic tissues, which may cause the high uptake regions of the lesion are spatially separated (Fig. 3). Previous studies have shown that ^18^F-FDG accumulates in inflamed lung lesions with activated inflammatory cells, particularly neutrophils [23]. Compared to tumor cells, inflammatory cells were more spatially separated. This might be the reason why the busyness and coarseness of benign lesions are significantly different from those of malignant lesions. Compared to early time-point images, tissues with high glycolysis, increased cell proliferation rate, and enhanced expression of hexokinase type-II and glucose transporter-1 may have an increased FDG uptake in tumor cells in delayed time-point imaging. At the same time, a longer distribution time also allows improved blood pool and urinary tract clearance of FDG and thus lower background activity. Therefore, on delayed time-point images, the images were less affected by blood perfusion, and the uptakes of normal tissues had washed out, which means that the inflammation lesions appear with more fineness in the delayed images and malignant lesions appear coarser. In addition, with the decay of the ^18^F-FDG in the tissue, delayed time-point images had higher noise than early time-point images, which led to a possible higher bias in SUV_max_ measurement.

Considering how these biological effects influence imaging properties, delayed image busyness might be more properly presenting the heterogeneity of lesions than early image busyness, thus increasing its discriminatory power between malignant and benign lesions as shown in this study.

This study used a retrospective cohort to prove that NGTDM texture features are a good predictor that can provide physicians with more information to supplement SUV_max_ in differentiating malignant from benign FDG-avid SPNs. Future studies should evaluate the accuracy of using NGTDM texture features in a larger prospective cohort from granuloma-endemic regions and whether the clinical decisions made based on those texture features actually improve clinical outcomes.

## Conclusions

NGTDM texture features extracted from the NGTDM were useful for diagnosing malignant and benign SPNs, especially the texture features from delayed PET/CT images. Improvement in discriminating benign from malignant nodules using SUV_max_ and visual interpretation can be achieved by adding busyness extracted from delayed PET/CT images. Based on our results, we recommend that NGTDM texture feature evaluation of delayed PET/CT scans is used as a predictor of SPN malignancy in clinical practice.

## Additional file

Additional file 1: Receiver operating characteristic (ROC) analysis was performed on SUVpeak and MATV. And the Pearson correlations test was performed to evaluate the correlation relationship between MATV and texture feature. (DOCX 38 kb)

## Data Availability

Please contact author for data requests.

## Abbreviations

FDG: Flourodeoxyglucose
NGTDM: Neighboring gray tone difference matrix
NPV: Negative prediction value
PPV: Positive prediction value
ROC: Receiver operation curve
SPN: Solitary pulmonary nodules
VOI: Volume Of Interest
