# Supplementary material for: Using neighborhood gray tone difference matrix texture features on dual time point PET/CT images to differentiate malignant from benign FDG-avid solitary pulmonary nodules
Source: Cancer Imaging. 2019 Aug 16;19:56. doi: 10.1186/s40644-019-0243-3 (PMC6697997; doi:10.1186/s40644-019-0243-3)
Supplement: Supplementary file 1 — Receiver operating characteristic (ROC) analysis was performed on SUVpeak and MATV. And the Pearson correlations test was performed to evaluate the correlation relationship between MATV and texture feature. (DOCX 38 kb) [file 40644_2019_243_MOESM1_ESM.docx]

| **Supplementary information**  **Materials and methods**  Receiver operating characteristic (ROC) analysis was performed on SUVpeak and MATV. The discriminant power of each index was evaluated using the areas under the ROC curves (AUC). The Pearson correlations test was performed to evaluate the correlation relationship between MATV and texture features. All statistical analyses were performed using SPSS 17.0 software. P < 0.05 was considered to indicate statistical significance.  **Results**  The ROC analysis (Table S1, Figure S1) showed that Early SUVpeak and Delayed SUVpeak worked similar with Early SUVmax and Delayed SUVmax, and the Early MATV and Delayed MATV were not a good predictor.  The correlation coefficients of volume and texture features were calculated (Table S2 and Table S3). The Pearson correlation test showed the SUVmax and texture features are correlated with MATV on both early (standard) and delayed time point PET images (P<0.05)，but the correlations coefficients is low.  **TABLES**  **Table S1. Area Under the ROC Curve**   \| Variable(s) \| Area \| \| --- \| --- \| \| Early SUVpeak \| 0.75 \| \| Early SUVmax \| 0.75 \| \| Delayed SUVpeak \| 0.74 \| \| Delayed SUVmax \| 0.74 \| \| Early MTAV \| 0.58 \| \| Delayed MTAV \| 0.59 \|   **Table S2. The Pearson Correlations Coefficient of MATV and indexes on early PET images** | | | | |
| --- | --- | --- | --- | --- | --- | --- | --- | --- | --- | --- | --- | --- | --- | --- | --- | --- | --- | --- |
|  | Early SUVmax | Early Coarseness | Early Contrast | Early Busyness |
| Early MTAV | 0.396 | 0.401 | -0.514 | 0.353 |

| **Table S3. The Pearson Correlations Coefficient of MATV and indexes on delayed PET images** | | | | |
| --- | --- | --- | --- | --- |
|  | Delayed SUVmax | Delayed Coarseness | Delayed Contrast | Delayed Busyness |
| Delayed MTAV | 0.380 | 0.313 | -0.440 | 0.305 |

**FIGURE**


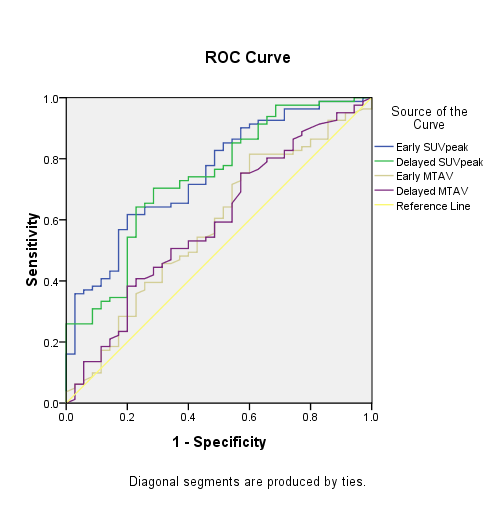


Figure S1. ROC curve of early SUVpeak, Delayed SUVpeak, Early MTAV and Delayed MTAV. The ROC analysis showed Early SUVpeak and Delayed SUVpeak worked similar with Early SUVmax and Delayed SUVmax, and the Early MATV and Delayed MATV were not a good predictor.
